# Endovascular treatment of fenestration of the posterior communicating artery with an aneurysm at the same site: case report and review of the literature

**DOI:** 10.3389/fradi.2025.1655243

**Published:** 2025-10-09

**Authors:** Yuxing Zheng, Antong Hu, Ziyun Gao

**Affiliations:** ^1^ The Second Affiliated Hospital, Jiangxi Medical College, Nanchang University, Nanchang, Jiangxi, China; ^2^ Department of Neurosurgery, The Second Affiliated Hospital, Jiangxi Medical College, Nanchang University, Nanchang, Jiangxi, China

**Keywords:** posterior communicating artery, fenestration, intracranial aneurysms, endovascular treatment, neuroradiology

## Abstract

**Background:**

The anatomical definition of fenestration in the posterior communicating artery (PCoA) has long been contentious. Previously reported cases exhibiting “dual-origin” characteristics more closely align with partial duplication, resulting in a lack of definitive clinical evidence for true fenestrations. This study presents the first globally reported case of a PCoA fenestration confirmed by multimodal imaging and co-occurring with an aneurysm at the same site, providing critical evidence for establishing imaging diagnostic criteria for fenestrations.

**Case presentation:**

A 65-year-old woman presented with persistent dizziness. Digital subtraction angiography (DSA) revealed a localized fenestration at the origin of the left PCoA, with a saccular aneurysm arising proximal to the fenestrated segment. Intraoperative 3D rotational angiography definitively characterized the fenestration as an interruption in a single vessel wall without parallel vascular structures (excluding partial duplication). The aneurysm was successfully treated via endovascular coil embolization, achieving Raymond-Roy Class I occlusion. No recurrence was observed at 12-month follow-up (mRS score 0).

**Conclusion:**

This study establishes the first imaging diagnostic criteria for PCoA fenestration, demonstrating that it can be distinguished from partial duplication by the key radiological feature of “single-vessel-wall interruption.” Embryologically, PCoA fenestration likely results from abnormal fusion of primitive embryonic vascular plexuses, with hemodynamic disturbance at the fenestration site identified as a critical mechanism for aneurysm formation. This case suggests the potential safety and efficacy of endovascular intervention proved safe and effective for managing intracranial aneurysms associated with arterial fenestration at the same location.

## 1 Introduction

Intracranial arterial fenestration is a rare congenital vascular variant with significant site-specific prevalence differences. Literature reports indicate the basilar artery (0.3%–6.0%) and anterior cerebral artery (0.1%–7.2%) as the most frequent fenestration sites (1–3), whereas fenestration of the posterior communicating artery (PCoA) occurs in merely 0.34% of cases (4). Four previously documented PCoA fenestration cases (5–8) exhibited separate dual origins, better categorized as partial duplications, suggesting the true incidence of PCoA fenestration is even lower. An angiographically confirmed case of true PCoA fenestration (without aneurysm) has also been documented in a curated online neurovascular atlas, supporting its existence as a distinct anatomical entity (9). Fenestrations of the PCoA with associated aneurysms are exceptionally rare. To our knowledge, this represents the first documented case of DSA-confirmed PCoA fenestration with an aneurysm, systematically elucidating its imaging characteristics, embryological basis, and clinical implications through literature review.

## 2 Case report

### 2.1 Clinical presentation

**Figure 1:**
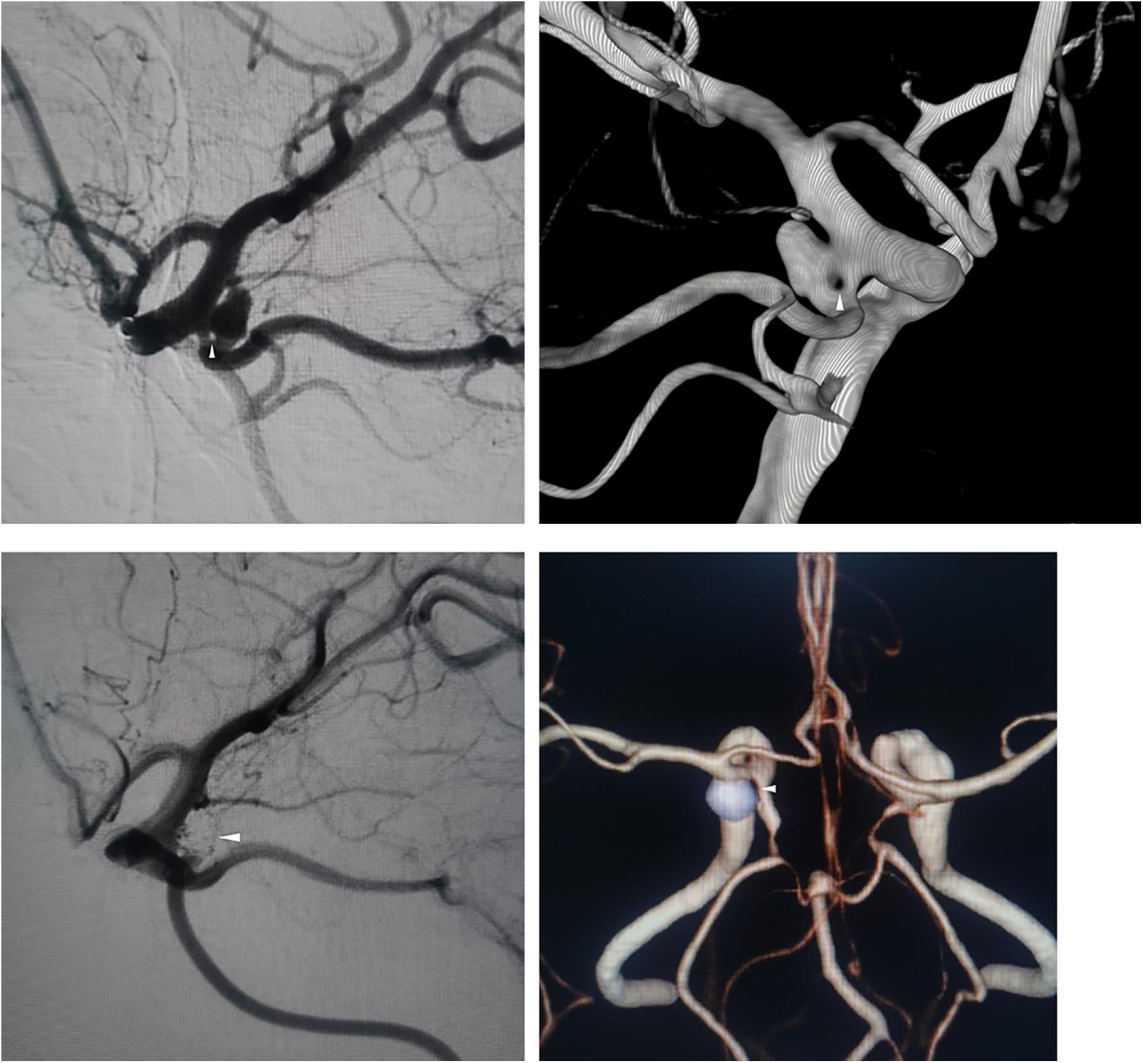
**(A)** Preoperative DSA shows left PCoA fenestration (arrow) and aneurysm. **(B)** 3D-RA confirms single-segment fenestration. **(C)** Postoperative DSA demonstrates complete aneurysm occlusion. **(D)** 12-month follow-up CTA confirms no recurrence.

A 65-year-old woman presented to a local hospital with chronic, persistent dizziness. There was no history of acute severe headache, meningismus, or decreased consciousness to suggest aneurysm rupture. Cranial computed tomography angiography (CTA) demonstrated a left posterior communicating artery (PCoA) aneurysm and internal carotid artery stenosis. Non-contrast CT showed no evidence of subarachnoid hemorrhage. Following 4 days of conservative observation, she was discharged but continued to experience intermittent dizziness without associated symptoms, prompting referral to our institution for definitive aneurysm management. Digital subtraction angiography (DSA) at our center confirmed a 0.8 mm fenestration in the left PCoA with a coexisting saccular aneurysm measuring 3.52 × 2.12 × 2.93 mm at the same location (Figure 1A). Three-dimensional DSA reconstruction further characterized the aneurysm morphology (Figure 1B). Her medical history included untreated hypertension for 3 years, with no history of diabetes, coronary artery disease, renal disorders, tobacco use, or alcohol consumption. Neurological examination revealed an alert and oriented patient (GCS 15) with pupils equal, round, and briskly reactive to light. A comprehensive cranial nerve examination was normal; specifically, she had no diplopia, ptosis, or disconjugate gaze to suggest oculomotor or other cranial nerve palsy. She exhibited full bilateral extremity strength (grade V), normal muscle tone, and no focal neurological deficits.

### 2.2 Indication for intervention

The decision to pursue endovascular treatment was multifactorial. Although the aneurysm was small, its location proximal to the fenestration—a site of inherent hemodynamic stress—raised concern for potential instability. Furthermore, the patient's persistent dizziness, in the absence of other explanatory causes, was clinically attributed to the aneurysm, warranting intervention to alleviate symptoms and prevent future rupture.

### 2.3 Clinical course and nursing management

The patient's clinical timeline, encompassing key diagnostic, interventional, and nursing care events, is summarized in Table 1.

**Table 1:** Timeline of relevant clinical events and interventions for the patient.

Time	Clinical events/interventions	Outcomes/patient response
Hospital day 1	Admitted due to persistent dizziness. Neurological examination performed.	GCS score 15, no focal neurological deficits. Non-contrast head CT: No acute hemorrhage.
Hospital day 2	Underwent digital subtraction angiography (DSA).	Revealed a 3.5 mm aneurysm at the fenestrated left posterior communicating artery (PCoA).
Hospital day 3	Preoperative nursing preparation: Patient education provided and surgical informed consent obtained.	Patient verbalized understanding of the procedure.
Hospital day 4	Underwent endovascular coil embolization. Transferred to the Neuro-ICU postoperatively.	Procedure successful (Raymond-Roy Class I occlusion). Vital signs stable.
Postoperative day 1 (POD #1)	Nursing care: Monitoring of vital signs q1h, puncture site assessment, pain management.	Patient alert and oriented. Denied pain. Puncture site clean and dry.
Postoperative day 2 (POD #2)	Transferred to general ward. Assisted with ambulation. Diet advanced to regular.	Reported occasional dizziness after ambulation. Diet tolerated well.
Postoperative day 3 (POD #3)	Discharged with medication and follow-up guidance.	mRS score 0. Reported no discomfort.
12-month follow-up	Follow-up CTA and outpatient clinic visit.	No aneurysm recurrence. mRS score 0.

GCS, glasgow coma scale; CT, computed tomography; DSA, digital subtraction angiography; PCoA, posterior communicating artery; Neuro-ICU, neurological intensive care unit; q1h, every 1 h; mRS, modified rankin scale; CTA, CT angiography.

### 2.4 Interventional treatment

Endovascular embolization was performed using a dual-microcatheter technique. An initial 4 × 10 cm framing coil was deployed, followed by sequential placement of four additional coils ranging from 2 × 4 cm to 1.5 × 1 cm. Through intentional coil protrusion into the aneurysmal limb of the fenestrated segment, parent vessel occlusion was achieved while preserving flow through the contralateral channel. Final angiography demonstrated complete aneurysm occlusion classified as Raymond-Roy Class I (Figure 1C).

### 2.5 Outcome and follow-up

Twelve-month postoperative CTA surveillance revealed no recurrence. The patient remained neurologically intact with a modified Rankin Scale score of 0 (Figure 1D).

## 3 Literature review methodology

A systematic search of PubMed and Embase databases (1980–2025) was conducted using the key terms “posterior communicating artery fenestration,” “fenestration,” and “intracranial aneurysm.” This yielded 8 cases reported as PCoA fenestrations (as summarized in Table 2). Critical analysis revealed that in four cases reported by Tripathi et al., Baba et al., Weiner et al., and Gunnal SA et al., both imaging and intraoperative findings demonstrated two separate vessels originating from distinct sites along the internal carotid artery with parallel courses converging into the posterior cerebral artery—consistent with partial duplication of the PCoA rather than true fenestration (10, 11).

**Table 2:** Literature review and characteristics of previously reported cases of pCoA fenestration or partial duplication.

Author (Year)	Sex/Age	Initial symptom/reason for study	Diagnostic method	Vascular origin	Aneurysm present	Accurate description	Case count
Yasargil (1984) (14)	2 Cadavers	Incidental finding	Anatomical	Single ICA origin	Yes	Fenestration	2
Tripathi et al. (2003) (5)	M/21	Right oculomotor nerve palsy	DSA	Dual ICA origins	No	Duplication	1
Baba et al. (2010) (6)	M/62	Physical examination	DSA/Surgical	Dual ICA origins	Yes	Duplication	1
Weiner et al. (2015) (7)	F/52	SAH	DSA/Surgical	Dual ICA origins	Yes	Duplication	1
Trandafilović M et al.(2016) (4)	2 Fetal Specimens	Analysis of fenestration/duplication incidence	Anatomical	Single ICA origin	No	Duplication	2
Gunnal SA et al. (2018) (8)	1 Cadaver	Study of PCoA variations	Anatomical	Dual ICA origins	No	Duplication	1
Current case	F/65	Dizziness	DSA	Single ICA origin	Yes	Fenestration	1

## 4 Discussion

### 4.1 Embryological basis

The posterior communicating artery (PCoA) originates from the primitive carotid-posterior cerebral arterial plexus during embryogenesis. Normally, this plexiform vascular network regresses into a single vessel by weeks 7–8 of gestation (12). Fenestration likely results from incomplete fusion of local vessel walls, creating a fenestrated segment within a single artery, whereas partial duplication manifests as dual-origin parallel vessels due to persistent plexiform structures (10).

### 4.2 Hemodynamic mechanism

Computational fluid dynamics (CFD) simulations demonstrate that fenestration malformations increase flow velocity gradients proximal to the fenestration site, inducing abnormal endothelial shear stress on the vascular wall (13). This mechanism is well-documented at more common fenestration sites, such as the basilar and anterior communicating arteries, where aneurysms form with significantly higher frequency compared to normal arterial segments (1–3). In this case, aneurysm formation proximal to the fenestration may be attributed to inherent structural fragility from aberrant embryological development compounded by postnatal hemodynamic stressors.

### 4.3 Clinical implications

In this case, intervention was advocated not only due to the aneurysm's presence at a high-risk morphological site but also because of the symptomatic presentation, which, although not definitively causal, justified preemptive treatment to eliminate a potential source of symptoms and avert rupture. For PCoA fenestration with co-occurring aneurysms, high-resolution 3D rotational angiography (3D-RA) is essential to differentiate fenestration from partial duplication and confirm the characteristic single-vessel fenestration morphology (10), thereby preventing misdiagnosis-related therapeutic errors. Treatment should prioritize preserving flow distal to the fenestration. Compared to stent-assisted coiling, the dual-microcatheter technique eliminates postoperative antiplatelet therapy-associated bleeding risks and is better suited for regions rich in perforating vessels (12). This approach is supported by literature reporting successful coil embolization of aneurysms at fenestration sites in more common locations, such as the basilar artery and anterior communicating complex (1, 3, 13). Long-term surveillance with periodic DSA or high-resolution MR angiography is mandatory to monitor flow dynamics in the contralateral vessel and fenestrated segment, mitigating risks of *de novo* aneurysm formation (13). While CTA is a practical and effective modality for routine monitoring of coil stability and aneurysm recurrence, periodic DSA or high-resolution MR angiography remains the gold standard for detailed hemodynamic assessment when available and clinically indicated.

## 5 Conclusion

This study establishes the inaugural imaging diagnostic criteria for posterior communicating artery (PCoA) fenestration, definitively distinguishing it from partial duplication through the pathognomonic radiological feature of single-vessel-wall interruption. Embryological analysis indicates that PCoA fenestration likely arises from aberrant fusion of primitive carotid-posterior cerebral arterial plexuses during vasculogenesis (12). Critically, computational hemodynamic modeling and clinical evidence converge to identify turbulent flow at the fenestration site as the primary driver for aneurysm formation. The presented case illustrates that endovascular coil embolization may be a safe and effective therapeutic strategy for aneurysms coexisting with such vascular variants, consistent with treatment outcomes for aneurysms at fenestrations in other locations (1, 3). Long-term surveillance remains essential given the inherent hemodynamic stresses in fenestrated segments. These findings provide a framework for risk stratification and management of this rare neurovascular entity.

## Data Availability

The datasets presented in this article are not readily available because of ethical and privacy restrictions. Requests to access the datasets should be directed to the corresponding author.
