# Supplementary material for: Endovascular treatment of fenestration of the posterior communicating artery with an aneurysm at the same site: case report and review of the literature
Source: Front Radiol. 2025 Oct 9;5:1655243. doi: 10.3389/fradi.2025.1655243 (PMC12545085; doi:10.3389/fradi.2025.1655243)
Supplement: Supplementary file 1 [file Table1.docx]

Supplementary Material

# Supplementary Tables

**Table 1: Literature Review and Characteristics of Previously Reported Cases of PCoA Fenestration or Partial Duplication**

| Author (Year) |  | Sex/Age | Initial  Symptom/Reason for Study | Diagnostic Method | Vascular Origin | Aneurysm Present | Accurate Description | Case Count |
| --- | --- | --- | --- | --- | --- | --- | --- | --- |
| Yasargil（1984）^11^ |  | 2 Cadavers | Incidental finding | Anatomical | Single ICA origin | Yes | Fenestration | 2 |
| Tripathi et al. (2003)^5^ |  | M/21 | Right oculomotor nerve palsy | DSA | Dual ICA origins | No | Duplication | 1 |
| Baba et al. (2010)^6^ |  | M/62 | Physical examination | DSA/Surgical | Dual ICA origins | Yes | Duplication | 1 |
| Weiner et al. (2015)^7^ |  | F/52 | SAH | DSA/Surgical | Dual ICA origins | Yes | Duplication | 1 |
| Trandafilović M et al.(2016)^4^ |  | 2 Fetal Specimens | Analysis of fenestration/duplication incidence | Anatomical | Single ICA origin | No | Duplication | 2 |
| Gunnal SA et al.（2018）^8^ |  | 1 Cadaver | Study of PCoA variations | Anatomical | Dual ICA origins | No | Duplication | 1 |
| Current case |  | F/65 | Dizziness | DSA | Single ICA origin | Yes | Fenestration | 1 |
